# Whole genome sequencing analysis to evaluate the influence of T2DM on polymorphisms associated with drug resistance in *M. tuberculosis*

**DOI:** 10.1186/s12864-022-08709-z

**Published:** 2022-06-24

**Authors:** Gustavo Adolfo Bermudez-Hernández, Damián Eduardo Pérez-Martínez, Carlos Francisco Madrazo-Moya, Irving Cancino-Muñoz, Iñaki Comas, Roberto Zenteno-Cuevas

**Affiliations:** 1 Health Sciences Doctoral Program. Health Sciences Institute, University of Veracruz, Jalapa, Mexico; 2 Biomedical Institute of Valencia IBV-CSIC, Valencia, Spain; 3 CIBER of Epidemiology and Public Health, Madrid, Spain; 4 Public Health Institute, University of Veracruz, Av. Luis Castelazo Ayala S/N, Col. Industrial Ánimas. Xalapa, A.P. 57, Veracruz, 91190 México; 5 Multidisciplinary Network of Tuberculosis Research, Veracruz, Mexico

**Keywords:** Diabetes, Tuberculosis, Drug resistance, Polymorphisms

## Abstract

**Background:**

Type 2 diabetes mellitus (T2DM) has been associated with treatment failure, and the development of drug resistance in tuberculosis (TB). Also, whole-genome sequencing has provided a better understanding and allowed the growth of knowledge about polymorphisms in genes associated with drug resistance. Considering the above, this study analyzes genome sequences to evaluate the influence of type 2 diabetes mellitus in the development of mutations related to tuberculosis drug resistance. *M. tuberculosis* isolates from individuals with (*n* = 74), and without (*n* = 74) type 2 diabetes mellitus was recovered from online repositories, and further analyzed.

**Results:**

The results showed the presence of 431 SNPs with similar proportions between diabetics, and non-diabetics individuals (48% vs. 52%), but with no significant relationship. A greater number of mutations associated with rifampicin resistance was observed in the T2DM-TB individuals (23.2% vs. 16%), and the exclusive presence of *rpoB*Q432L, *rpoB*Q432P, *rpoB*S441L, and *rpoB*H445L variants. While these variants are not private to T2DM-TB cases they are globally rare highlighting a potential role of T2DM. The phylogenetic analysis showed 12 sublineages, being 4.1.1.3, and 4.1.2.1 the most prevalent in T2DM-TB individuals but not differing from those most prevalent in their geographic location. Four clonal complexes were found, however, no significant relationship with T2DM was observed. Samples size and potential sampling biases prevented us to look for significant associations.

**Conclusions:**

The occurrence of globally rare rifampicin variants identified only in isolates from individuals with T2DM could be due to the hyperglycemic environment within the host. Therefore, further studies about the dynamics of SNPs’ generation associated with antibiotic resistance in patients with diabetes mellitus are necessary.

**Supplementary Information:**

The online version contains supplementary material available at 10.1186/s12864-022-08709-z.

## Introduction

Tuberculosis (TB) maintains a severe impact on global public health; according to the World Health Organization (WHO), in 2020 it caused 1.5 million deaths, and 10 million people sickened. One of the factors associated with the disease´s high mortality is the development of drug resistance. In 2018, approximately 500,000 individuals developed resistance to rifampicin, and from this patient’s group, 82% developed multi-drug resistance [1].

Studies have observed an increased risk of treatment failure, and the development of drug resistance in people with type 2 diabetes mellitus (T2DM). However, the impact of this condition on the development of drug resistance remains unclear [2–4]. A possible explanation may lie in the altered pharmacokinetics of T2DM-TB cases leading to heterogenous drug exposure during treatment [3].

Whole-genome sequencing (WGS) has opened the possibility to increase our understanding of drug resistance mechanisms. Particularly, the identification of the presence of polymorphisms in target genes associated with the performance of anti-tuberculosis drugs will aid in the development of the predictive diagnosis of drug resistance in TB [5].

Considering all the above, the present study aims to characterize the polymorphisms associated with drug resistance, and to determine the influence of diabetes in the occurrence of these mutations by analyzing whole genome sequences of *M. tuberculosis* (Mtb) from individuals with TB, and T2DM-TB binomial.

## Results

### Patient characteristics

From more than a 1 million Mtb WGS currently available in the repositories analyzed, only 827 genomes had clinical information related to the resistance profile and confirmation of the presence or absence of T2DM. From this group, only 74 genomes from individuals with T2DM had the required sequencing depth, lineage, and coverage to be included in the study. An additional set of 74 WGS from individuals with drug resistance and without T2DM were retrieved and randomly included to match both study groups. Individuals carrying the 148 Mtb strains were mostly male 84 (57.4%), with a mean age of 47 ± 13 years at the time of sputum sample collection.

Patients came from nine countries, mainly Georgia with 36 participants (24%), Mexico, 34 (22.9%), Moldova, 20 (13.5%), and Belarus with 15 (10.1%). According to the type of resistance, four groups of isolates were identified: 40 were monoresistant (MR) (27%), 18 polyresistant (POL) (12.1%), 62 multidrug-resistant (MDR) (41.8%), and 28 extensively drug resistant (XDR) (18.9%) (Table 1).

**Table 1:** Epidemiological characteristics of the sample

	**T2DM isolates**^**a**^	**TB isolates**^**b**^	**Total**
	**(*n* = 74)**	**(*n* = 74)**	**(*n* = 148)**
**Sex**			
Male	44(59%)	40 (54%)	84(57.4%)
Female	30 (41%)	34 (45%)	64(42.6%)
**Age**			
**Mean (± SD)**	50 (± 10)	42 (± 15)	47 (± 13)
**Country**			
Georgia	8 (10.8%)	28 (37%)	36 (24.3%)
Mexico	23 (31%)	11 (14.8%)	34 (23%)
Moldova	6 (8.1%)	14 (18.9%)	20 (13.5%)
Belarus	4 (5.4%)	11 (14.8%)	15 (10.1%
Romania	2 (2.7%)	10 (13.5%)	12 (8.1%)
Peru	11 (14.8%)	0 (0.0%)	11 (7.4%)
Indonesia	9 (12.1%)	0 (0.0%)	9 (6.1%)
Spain	8 (10.8%)	0 (0.0%)	8 (5.4%)
Azerbaijan	3 (4%)	0 (0.0%)	3 (2%)
**Resistance type**^**c**^			
MR	20 (27%)	20 (27%)	40 (27%)
POL	9 (12.1%)	9 (12.1%)	18 (12.1%)
MDR	31 (48.8%)	31 (48.8%)	62 (41.8%)
XDR	14 (18.9%)	14 (18.9%)	28 (18.9%)
**Drug-resistance**			
Isoniazid	58 (67.5%)	55 (74.3%)	113 (76.3%)
Rifampicin	50 (67.5%)	53 (71.6%)	103 (69.5%)
Streptomycin	21 (28.3%)	42 (56.7%)	63 (42.5%)
Ethambutol	27 (36.4%)	37 (50%)	64 (43.2%)
Pyrazinamide	27 (36.4%)	24 (32.4%)	51 (34.4%)
Amikacin	19 (25.6%)	30 (40.5%)	49 (33.1%)
Fluoroquinolones	16 (21.6%)	17 (22.9%)	33 (22.3%)

^a^T2DM: Host with diabetes mellitus type 2 and tuberculosis. ^b^TB Isolates: Host without diabetes mellitus type 2 and tuberculosis. ^c^MR: mono-resistant tuberculosis, *POL* poly-resistant tuberculosis, *MDR* multidrug-resistant tuberculosis, *XDR* extensively drug resistant tuberculosis

### Characterization of variants associated with resistance

Genotypic resistance in the isolates was observed for several drugs, with the highest proportion of resistance to isoniazid (INH) with 113 isolates (76.3%), and rifampicin (RIF) including 103 strains (69.5%), followed by ethambutol (EMB) 64 (43.2%), streptomycin (STR) 63 (42.5%), and pyrazinamide (PZA) in 51 strains (34.4%). Genotypic resistance to second-line drugs had a lower representation with only 49 sequences resistant to amikacin (AMK) (33.1%), and 33 (22.3%) to fluoroquinolones (FQ). Among these drugs, only STR showed a significant difference in the number of WGS with this type of resistance with 21 (28.3%) isolates in the T2DM group vs 42 (56.7%) in the TB group (*p* = 0.00048).

During this study were identified 431 SNPs confirmed to be associated with resistance [6] in the diabetic, and non-diabetic groups; 207 (48%) vs 224 (52%) respectively. Only 23 (12 [2.7%] vs 11 [2.5%]) were classified as non-fixed SNPs. The mean number of SNPs by isolate was 2.79 vs 3.02 with a 2.7 vs 2.8 standard deviation, with no significant association in the distribution between groups (*p* = 0.4413). Regarding the distribution of polymorphisms by type of resistance, the sequences from individuals with diabetes presented a higher frequency in isolates with monoresistance, 21 (7%) vs 20 (6.9%) (*p* = 0.7073), and polyresistant, 25 (8.8%) vs 14 (4.6%) (*p* = 0.4221). In contrast, lower frequencies of polymorphisms were identified in isolates classified as MDR, 136 (47.7%) vs 159 (52.0%) (*p* = 0.2659), and XDR, 104 (36.5%) vs 112 (36.6%) (*p* = 0.9281).

Fifty-six high confidence resistance-related variants were identified, from which, 16 had a proportion greater than 1% in the dataset. Genotypic resistance was given by genomic variants associated with anti-tuberculosis drugs, and mainly concentrated in genes associated with this property. Resistance to isoniazid was given by in 58 isolates (78.3%) from the T2DM group, vs 55 on the isolates (71.6%) with only TB (*p* = 0.3368), rifampicin 50 (67.5%) vs 53 (71.6%) (*p* = 0.2874) and ethambutol 27 (36.4%) vs 37 (50%) (*p* = 0.2753).

Three mutations were observed with a frequency greater than 15% in both groups; *katG* S315T was found in 91 sequences (61.4%), and *fabG1-inhA* in 62 (41.8%); these were the only genes associated with resistance to isoniazid observed in the dataset. These mutations were followed by *rpoB* S450L in 58 isolates (39.1%) (Table 2). Nevertheless, no differences on these mutations were observed in the groups. However, when comparing between the groups, no significant differences were observed in the variants associated with resistance to isoniazid (*p* = 0.9445) and (*p* = 0.6253), respectively, nor for rifampicin (*p* = 0.9675).

**Table 2:** Allele frequency of SNPs associated with drug resistance^a^

Antibiotic resistance	Gene	Aminoacid change	Frequency T2DM^b^ (*n* = 74) n(%)	Frequency TB^c^ (*n* = 74) n(%)	Frequency Total (*n* = 148) n(%)
Isoniazid	*katG*	S315T	44 (59.4%)	47 (63.5%)	91 (61.4%)
	*fabG1-inhA*	c15t	28 (37.8%)	34 (45.9%)	62 (41.8%)
Rifampicin	*rpoB*	S450L	28 (37.8%)	30 (40.5%)	58 (39.1%)
	*rpoB*	H445D	5 (6.7%)	11 (14.8%)	16 (10.8%)
	*rpoB*	D435Y	2 (2.7%)	6 (8.1%)	8 (5.4%)
	*rpoB*	H445L	4 (5.4%)	0	4 (2.7%)
	*rpoB*	S441L	1 (1.3%)	0	1 (0.6%)
	*rpoB*	Q432P	1 (1.3%)	0	1 (0.6%)
	*rpoB*	Q432L	1 (1.3%)	0	1 (0.6%)
	*rpoC*	V483G	8 (10.8%)	10 (13.5%)	18 (12.1%)
	*rpoC*	V483A	3 (4.0%)	2 (2.7%)	5 (3.38%)
Streptomycin	*rpsL*	K88R	7 (9.4%)	15 (20.2%)	22 (14.8%)
	*rpsL*	K43R	4 (5.4%)	14 (18.9%)	18 (12.1%)
Ethambutol	*embB*	M306I	12 (16.2%)	9 (12.1%)	21 (14.1%)
	*embB*	Q497R	5 (6.7%)	12 (16.2%)	17 (11.4%)
	*embB*	S297A	4 (5.4%)	9 (12.1%)	13 (8.7%)
Pyrazinamide	*pncA*	D49G	5 (6.7%)	6 (8.1%)	11 (7.4%)
	*pncA*	L120P	7 (9.4%)	2 (2.7%)	9 (6.8%)
Fluoroquinolone	*gyrA*	A90V	4 (5.4%)	6 (8.1%)	10 (6.7%)
	*gyrA*	D94G	4 (5.4%)	3 (4.0%)	8 (5.4%)

^a^SNPs with a proportion greater than 3% in the sample (*n* = 148), ^b^T2DM: Host with diabetes mellitus type 2 and tuberculosis

^c^TB: Host without diabetes mellitus type 2 and tuberculosis

In only two SNPs was detected a difference in a greater proportion than 10% between diabetic, and non-diabetic groups; these were, *rpsL* K88R (9.4% vs 20.2%), and *rpsL* K43R (5.41% vs 18.9%), both related with resistance to STR. Regarding rifampicin resistance, 22 different *rpoB* variants were observed between the T2DM and TB groups, 13 (23.2%) vs. 9 (16%), respectively; four mutations were identified exclusively in isolates from T2DM group; *rpoB* H445L (observed in four isolates) and *rpoB* Q432L*, rpoB* Q432P and *rpoB* S441L (detected in only one isolate each) (Table 2). The *rpoB* S450L variant was identified in clonal complexes C2 and C3; whereas, *rpoB* H445D and *rpoB* H445L were observed in C1 and *rpoB* D435Y in C2.

Likewise, it was found that in 20 isolates carrying the *rpoB* S450L variant also had the compensatory mutation *rpoC* V483A/V483G. Among these, eight isolates (10.8%) from patients with T2DM carried two mutations, two *rpoC* V483A, and six *rpoC* V483G, meanwhile, in 12 strains (16.2%) from the non-diabetic group these mutations were also observed, 2 *rpoC* V483A, and 10 *rpoC* V483G.

### Phylogenetic characterization

Phylogenetic analysis of the isolates showed that 148 isolates were included in 12 sublineages of L4, being the most frequent 4.3.3(LAM9) with 31 sequences (21%), and 4.10 (PGG3) with 29 (20%), followed by 4.1.2.1 (Haarlem) with 25 (16%), and 4.2.1 (Ural) with 24 (16%). The LAM9 (22%), Ural (20.9%), and Haarlem (19%) sublineages showed the highest frequency of MDR isolates, on the other hand, LAM9 concentrated the highest proportion of XDR tuberculosis (43%).

Regarding the phylogenetic distribution based on diabetes comorbidity, differences in the prevalence of some sublineages were observed. Sublineages 4.1.1.3 (X3/X1), 13% vs 3%, and 4.1.2.1 (Haarlem), 24% vs 9%, were predominantly found in isolates from patients with T2DM, respectively. By comparison, the sublineages 4.3.3 (LAM9), 28% vs. 14%, 4.10 (PGG3), 26% vs. 14%, and 4.2.1 (Ural), 23% vs. 9%, were found in a higher proportion within non-diabetic individuals (Table 3).

**Table 3:** Mtb L4 sublineages identified by WGS

Sublineage	Code	T2DM isolates^a^	TB isolates^b^	Total
		**(*n* = 74)**	**(*n* = 74)**	**(*n* = 148)**
PGG3	4.10	10 (13.5%)	19 (25.6%)	29 (19.5%)
X	4.1.1	3 (4.0%)	2 (2.7%)	5 (3.3%)
X2	4.1.1.1	0 (0.0%)	1 (1.3%)	1(.06)
X3/X1	4.1.1.3	10 (13.5%)	2 (2.7%)	12 (8.1%)
T1;H1	4.1.2	1 (1.3%)	2 (2.7%)	3 (2%)
Haarlem	4.1.2.1	18 (24.3%)	7 (9.4%)	25 (16.8%)
Ural	4.2.1	7 (9.4%)	17 (22.9%)	24 (16.2%)
LAM3	4.3.2	5 (6.7%)	0 (0.0%)	5 (3.3%)
LAM9	4.3.3	10 (13.5%)	21 (28.3%)	31 (20.9%)
LAM1	4.3.4.1	3 (4.0%)	1 (1.3%)	4 (2.7%)
LAM11	4.3.4.2	1 (1.3%)	4 (5.3%)	5 (3.3%)
S	4.4.1	1 (1.3%)	0 (0.0%)	1(.06)

^a^T2DM: Host with diabetes mellitus type 2 and tuberculosis

^b^TB Isolates: Host without diabetes mellitus type 2 and tuberculosis

According to the drug resistance profile, 40% of the MR isolates belonged to the 4.10 (PGG3) sublineage, and 33.3% of the POL isolates were found in the 4.1.2.1 (Haarlem) sublineage. The highest proportion of MDR sequences was classified into three sublineages: 19.3% in 4.1.2.1 (Haarlem), 20.9% in 4.2.1 (Ural), and 22.5% in 4.3.3 (LAM9). XDR isolates were mainly classified in the 4.2.1 (Ural) sublineage 21.4%, and 4.3.3 (LAM9) 42.8%.

Phylogenetic analysis in the 148 isolates, with a 12 SNPs cut-off point, identified 36 isolates (24%) grouped in four clonal complexes (C): C1, with sublineage 4.3.3 (LAM9), composed of five isolates, all of them from the TB group, and from Belarus; C2, with lineage 4.2.1 (Ural), which includes 17 sequences, six T2DM vs 11 TB, 88% originating from Moldova; C3, with sublineage 4.1.1.3 (X3/X1), composed of nine isolates, six T2DM vs three TB) all from Mexico and C4 with sublineage 4.10 (PGG3), which includes five sequences from Georgia.

**Fig. 1:**
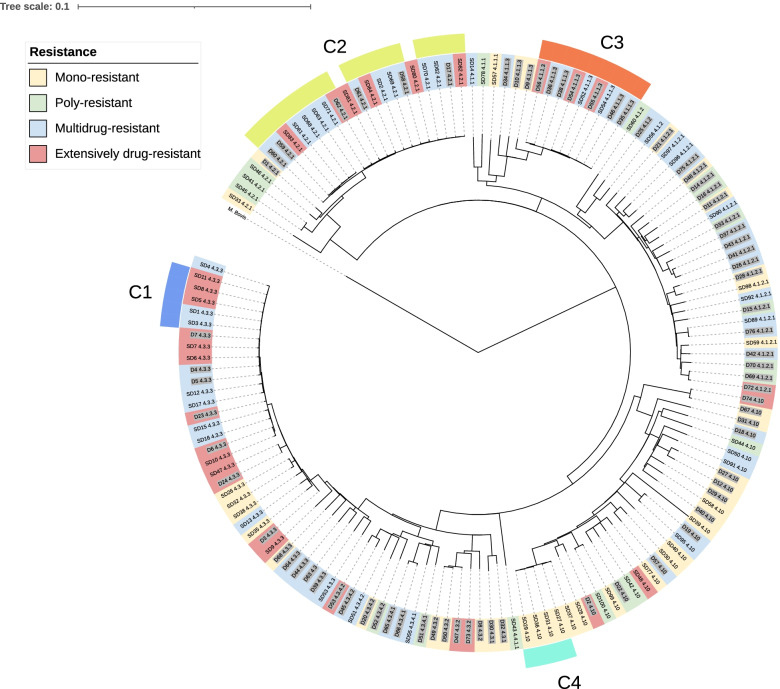
Phylogenetic tree rooted with M. bovis showing isolates lineages and the 4 clonal complexes found with a threshold of 12 SNPs. C1: lineage 4.3.3 (LAM9), C2: lineage 4.2.1 (Ural), C3: lineage 4.1.1.3 (X3/X1) and C4: lineage 4.10 (PGG3). Labels of patients with T2DM are highlighted in grey. Legend shows resistance classification. The scale represents the number of substitutions per site for each position of the alignment

A pattern of six mutations was observed in all C1 isolates: *katG S315T*, *rss (position 1,472,359)*, *embA* (position 4,243,221*)*, *embB* Q497R*, pncA* D49G, and *rpoB* H445D. Similarly, C2 included 14 sequences (82.3%), and shared the *katG S315T*, *fabG1-inhA, rpoB* S450L, and *rpsL* K88R mutation pattern, all these isolates came from Moldova. However, no relationship between diabetes comorbidity, and the presence of these variant patterns were identified. (Fig. 1).

The occurrence of rifampicin resistance-associated variants identified only in patients with T2DM had a heterogeneous phylogenetic distribution suggesting that they are not driven by geographically prevalent strains. The variants observed in a single isolate were *rpoB* S441L, and *rpoB* Q432P, belonging to sublineage 4.10 (PGG3), whereas *rpoB* Q432L, was observed in another isolate, and was found in sublineage 4.3.4.2 (LAM11). On the other hand, the *rpoB* H445L variant was observed in four isolates from three different sublineages 4.1.2 (T1;H1), 4.3.3 (LAM9), and 4.3.4.2 (LAM11).

## Discussion

Drug resistance generation in Mtb is caused by chromosomal mutations resulting in clones that proliferate, and subsequently fixate on the host [7]. The mutation rate of Mtb is low with an estimated 0.3–0.5 SNPS changes per year [8, 9], but this condition seems to be affected by the internal environment of the host, and by the pharmacokinetic variability of the plasma concentration of anti-tuberculosis drugs [10]. In this study, genome sequences of Mtb from individuals with drug resistant tuberculosis, and with or without T2DM, were analyzed to determine the impact of T2DM on the generation of drug resistance, given that diabetes increases the risk of developing drug resistance, and multidrug resistance [2–4, 11], also modifies the host’s internal environment by promoting the generation of reactive oxygen species in mycobacteria [12], which could lead to changes in the mutation rate in these subjects.

The polymorphism analysis showed that the most frequent variants were associated with the first-line drugs, isoniazid, and rifampicin. Mutations in the *katG* S315T and *fabG1-inhA* genes showed the highest proportions in both groups of individuals, this is consistent with previous studies [13, 14], and could be due to the fact that these variants are the first to appear in the genome of resistant strains from different parts of the world [15].

In the T2DM group four specific *rpoB* gene variants were found: H445L, S441L, Q432P, and Q432L. These variants have been reported in studies where comorbidities are not specified [16–19]. According to the recent Catalogue of mutations related with drug resistance in Mycobacterium tuberculosis complex of WHO, these mutations have a low prevalence (1.17%, 0.26%, 0.29% and 0.21% respectively), and have been classified as SNPs associated with phenotypic resistance against rifampicin [20]. These variants were found in four different sublineages: 4.10 (PGG3), 4.3.4.2 (LAM11), 4.1.2 (T1; H1) and 4.3.3 (LAM9). Only two isolates with the H445L variant were found in a clonal complex (C1), which could indicate that remaining mutations were not acquired through clonal transmission but were the product of processes specific of the host, possibly by consequence to the complications in the drug treatment in T2DM individuals, and partially explaining the increased risk for the development of drug resistance [2–4]. Also with relation to RIF resistance, the *rpoB Q432L,* and *rpoB Q432P* mutations have been identified in isolates with a high minimum inhibitory concentration (MIC), while *rpoB S441L,* and *rpoB H445L* showed medium, and low MIC respectively [18]. Likewise, pharmacokinetics studies have found that individuals with T2DM had 25% lower average plasma concentration, and an increase in time to reach the maximum concentration of rifampicin [3]. The *rpoB* Q432L and *rpoB* S441L variants, have been associated with a moderate fitness cost in in vitro studies [21], the previously mentioned changes in drug distribution could favor the proliferation of bacterial population carrying these mutations.

As explained above, the specific occurrence of these mutations in the T2DM group could be due to poor glycemic control that alters tissue perfusion, and rifampicin pharmacokinetics; however, the sample size in which we found these variants prevented us from looking for associations between the groups. Further studies with a larger number of individuals are needed to determine whether certain variants in the rifampicin resistance determining region in subjects with diabetes could be due to internal environmental conditions resulting from a hyperglycemic condition.

The phylogenetic analysis showed heterogeneity in the 12 sublineages found in both groups of individuals. The 4.1.1.3 (X3/X1), and 4.1.2.1 (Haarlem) sublineages included the higher number of isolates coming from individuals with T2DM. In particular, the largest difference in the isolates’ proportion between the groups was observed in the X3/X1 lineage, this could be linked to the country of origin of these isolates, since 83% came from Mexico. A similar situation happened with Haarlem sublineage, where 66% of the isolates from this sublineage, came from Mexico, and Indonesia. A total of 33 isolates were grouped into four clonal complexes, of which only C3, was composed of sublineage 4.1.1.3 (X3/X1) and showing a higher proportion of isolates from individuals with T2DM (six TB-T2DM vs. three TB). The high proportion, and differences observed in the distribution of isolates between diabetic, and non-diabetic individuals in the lineages from these countries could be explained with the fact that Mexico, and Indonesia are among the top 3 places in the worldwide prevalence of T2DM [22].

It has been described that the phylogenetic sublineages of *M. tuberculosis* complex are associated with specific geographic regions [23, 24]; therefore, the fact that diabetes was found with higher proportions in certain sublineages could be a consequence of the disease burden in the specific countries; moreover, although the relationship between lineage and glycemic level has not been characterized, a possible association between dysglycemia, and the distribution of Haarlem strains has been recently described [25]. To establish a clearer relationship, are required studies incorporating a larger number of WGSs from individuals with T2DM from different sublineages.

The strength of this study lies in the fact that, to the best of our knowledge, there are no studies using WGS to analyze the impact of T2DM on the emergence of SNPs associated with drug resistance in L4 isolates the largest Mtb lineage distributed globally [26]. Furthermore, the main limitation was the lack of information about comorbidities in genomic sequences of *M. tuberculosis* isolates available in repositories. From the 827 drug resistant isolates initially identified, only 74 fulfilled the criteria to be included in the TB-T2DM group, this stands out the need to include information related to comorbidity in the metadata available in the genome repositories. This would be of great help to improve further analysis of genomes, also to evaluate the specific effect of the host in the development of drug resistant in TB.

Finally, the lack of differences in the polymorphisms between the T2DM-TB, and TB groups could be due to the fact that these sequences come from isolates that had already been diagnosed with a resistance profile, as evidenced by the high proportion of fixed SNPs identified in the sample [27], which hinders the identification of the time of appearance of the resistance mutations, and the generation of these changes as consequence of the internal environment caused by type 2 diabetes mellitus in the host.

## Conclusions

With this study it was possible to provide preliminary information about the impact of T2DM on drug resistance in whole genome sequences of *M. tuberculosi*s, the results showed no significant association among the number of polymorphisms associated with resistance and the presence of T2DM. However, four variants in *rpoB* were observed only in individuals with T2DM. Undoubtedly, further studies about the dynamics of SNPs’ generation associated with antibiotic resistance in patients with diabetes mellitus are necessary, the results will have an important effect on the establishment of new therapeutic, attention, and control models for tuberculosis in the context of T2DM.

## Methods

### Sample selection

A search for whole genome sequences (WGSs) of Mtb from patients with drug resistance, and with or without T2DM was carried out in public databases; GenBank of the National Center for Biotechnology Information, the European Nucleotide Archive, and TB Portals of the National Institute of Health. In addition, 8 Mtb sequences were kindly provided by Dr. Iñaki Comas Espadas from Centro de Investigaciones Biomédicas de Valencia, Spain (manuscript in preparation). Only the WGS that incorporate the following information in the metadata associated were included in the study: presence/absence of a diagnosis of diabetes in the patient, phenotypic and genotypic profile of drug resistance, coverage greater than 99%, depth greater than 100X, and belonging to lineage 4 (L4). Furthermore, those sequences were grouped according to host characteristics into two groups: 1) Host with TB drug resistant, and diabetes mellitus type 2 (T2DM), and 2) Host with TB drug resistant, and without diabetes mellitus type 2.

Accession numbers of genomes selected are available in the Additional file 1.

### Bioinformatic analysis

The bioinformatic analysis was carried out using a previously validated pipeline (http://tgu.ibv.csic.es/?page_id=1794) [28, 29]. The workflow was composed by four sections: 1) Read cleaning contamination and removal, was done by means of Trimmomatic v0.36, Kraken v0.10.5 and Seqtk v1.3; 2) Mapping, and coverage calculation, with BWA v0.7.12, and Bedtools v2.26; 3) Variant (SNPs and indels) calling was carried out by using Samtools v1.3.1, VarScan v2.3.7, and GATK v 4.0.2.1 and, 4) Variant filtering performed by using a customed Python code.

Genomic variants that were present in at least 20 reads, and at ≥ 90% of frequency within each isolate were used to detect phylogenetic mutations and confirm the belonging to L4 and sublineage inclusion. Variants (InDels and SNPs) with less than 10 × were classified as low-coverage variants and were discarded from analyses. In addition, SNPs detected in high density variant regions were removed. We defined high-density regions whether we found > 3 SNPs in a window of 10 bp.

Variants with at least 10 reads at ≥ 10% to ≤ 90% frequency were called non fixed-SNP, and used to detect antibiotic resistance, and to confirm or predict the drug resistant profile. To determine drug resistance a combination of previously published mutation catalogs was used [30, 31]. The MTBC lineages were identified by matching fixed SNPs (those with a frequency of > 90%) to specific phylogenetic positions as described in other publications [26, 32].

The complete analysis pipeline can be found in the next Gitlab repository: https://gitlab.com/tbgenomicsunit/ThePipeline

### Phylogenetic analysis

The filtered reads (those belonging only to MTBC) were mapped against an ancestral reference genome and the polymorphisms were extracted. Identified mutations, INDELS (insertions and deletions) and SNPs were called if they were found in at least 10 reads and with a minimum quality of 20. SNPs were classified into two types, according to their frequency. Fixed SNPs (those detected at a minimum frequency of 90%) were used to classify samples into lineages, and to obtain the final alignment for the detection of clonal complexes, while variable SNPs (those detected at a frequency of 10–90%) were used to detect resistances. In order to avoid a false positive variant calling, SNPs annotated in PE/PPE/PGRS and phage genes were removed. In addition, SNPs (both fixed and variable) detected within insertion sequences, INDELS and high-density regions (> 3 SNPs en 10 bp) were discarded from analyses.

The phylogenetic tree was inferred from the resulting maximum likelihood SNP alignment in RAxML v8.2.4, by applying the General Time Reversible model of nucleotide substitution with the gamma distribution (GTRGAMMA) as used in other studies [28]. A 12 SNPs cut-off point was used to determine clonal complexes as recommended in previous studies [33, 34]. Phylogenetic tree visualization was done with iTOL v5 (https://itol.embl.de) [35].

### Statistical analysis

The data collected were summarized using descriptive statistics, to identify the differences in polymorphisms between the groups, the chi-square test, and spearman correlation were performed using SPSS v.25 software. A *p*-value < 0.05 was considered statistically significant.

## Supplementary Information

**Additional file 1. **Accession numbers of genomes and databases.

## Data Availability

The datasets supporting the conclusions of this article are available in the BioSample (https://www.ncbi.nlm.nih.gov/biosample/), ENA (https://www.ebi.ac.uk/ena/browser/home) and Tb portals (https://tbportals.niaid.nih.gov/) repositories. The accession numbers of the genomes selected for this study can be found in the Additional file 1.

## Abbreviations

T2DM: Type 2 diabetes mellitus
TB: Mycobacterium tuberculosis
WGS: Whole-genome sequencing
SNP: Single nucleotide polymorphism
MR: Monoresistant
XDR: Extensively drug resistant
POL: Polyresistant
MDR: Multidrug-resistant
INH: Isoniazid
RIF: Rifampicin
EMB: Ethambutaol
STR: Streptomycin
PZA: Pyrazinamide
AMK: Amikacin
FQ: Fluoroquinolones
L4: Lineage 4
MIC: Minimum inhibitory concentration

## References

[1] WHO. Global tuberculosis report 2018. Geneva: World Health Organization; 2018.

[2] Kornfeld H, Sahukar SB, Procter-Gray E, Kumar NP, West K, Kane K, Natarajan M, Li W, Babu S, Viswanathan V (2020). Impact of diabetes and low body mass index on tuberculosis treatment outcomes. Clin Infect Dis.

[3] Alfarisi O, Mave V, Gaikwad S, Sahasrabudhe T, Ramachandran G, Kumar H, Gupte N, Kulkarni V, Deshmukh S, Atre S (2018). Effect of diabetes mellitus on the pharmacokinetics and pharmacodynamics of tuberculosis treatment. Antimicrob Agents Chemother.

[4] Yoon YS, Jung J-W, Jeon EJ, Seo H, Ryu YJ, Yim J-J, Kim YH, Lee B-H, Park YB, Lee BJ (2017). The effect of diabetes control status on treatment response in pulmonary tuberculosis: a prospective study. Thorax.

[5] Schleusener V, Köser CU, Beckert P, Niemann S, Feuerriegel S (2017). Mycobacterium tuberculosis resistance prediction and lineage classification from genome sequencing: comparison of automated analysis tools. Sci Rep.

[6] Programme GT: Catalogue of mutations in Mycobacterium tuberculosis complex and their association with drug resistance. In.: World Heatlh Organization; 2021.

[7] Al-Saeedi M, Al-Hajoj S (2017). Diversity and evolution of drug resistance mechanisms in Mycobacterium tuberculosis. Infection and drug resistance.

[8] Hakamata M, Takihara H, Iwamoto T, Tamaru A, Hashimoto A, Tanaka T, Kaboso SA, Gebretsadik G, Ilinov A, Yokoyama A (2020). Higher genome mutation rates of Beijing lineage of Mycobacterium tuberculosis during human infection. Sci Rep.

[9] Takiff HE, Feo O (2015). Clinical value of whole-genome sequencing of Mycobacterium tuberculosis. Lancet Infect Dis.

[10] Allue Guardia A, Garcia JI, Torrelles JB (2021). Evolution of drug-resistant Mycobacterium tuberculosis strains and their adaptation to the human lung environment. Front Microbiol.

[11] Perez-Navarro LM, Restrepo BI, Fuentes-Dominguez FJ, Duggirala R, Morales-Romero J, López-Alvarenga JC, Comas I, Zenteno-Cuevas R (2017). The effect size of type 2 diabetes mellitus on tuberculosis drug resistance and adverse treatment outcomes. Tuberculosis.

[12] Ferlita S, Yegiazaryan A, Noori N, Lal G, Nguyen T, To K, Venketaraman V (2019). Type 2 diabetes mellitus and altered immune system leading to susceptibility to pathogens, especially Mycobacterium tuberculosis. J Clin Med.

[13] Lempens P, Meehan CJ, Vandelannoote K, Fissette K, de Rijk P, Van Deun A, Rigouts L, de Jong BC (2018). Isoniazid resistance levels of Mycobacterium tuberculosis can largely be predicted by high-confidence resistance-conferring mutations. Sci Rep.

[14] Charoenpak R, Santimaleeworagun W, Suwanpimolkul G, Manosuthi W, Kongsanan P, Petsong S, Puttilerpong C (2020). Association Between the Phenotype and Genotype of Isoniazid Resistance Among Mycobacterium tuberculosis Isolates in Thailand. Infection and drug resistance.

[15] Manson AL, Cohen KA, Abeel T, Desjardins CA, Armstrong DT, Barry CE, Brand J, Chapman SB, Cho S-N, Gabrielian A (2017). Genomic analysis of globally diverse Mycobacterium tuberculosis strains provides insights into the emergence and spread of multidrug resistance. Nat Genet.

[16] Ng KC, Meehan CJ, Torrea G, Goeminne L, Diels M, Rigouts L, de Jong BC, André E (2018). Potential application of digitally linked tuberculosis diagnostics for real-time surveillance of drug-resistant tuberculosis transmission: validation and analysis of test results. JMIR Med Inform.

[17] Bhembe NL, Nwodo UU, Govender S, Hayes C, Ndip RN, Okoh AI, Green E (2014). Molecular detection and characterization of resistant genes in Mycobacterium tuberculosis complex from DNA isolated from tuberculosis patients in the Eastern Cape province South Africa. BMC Infect Dis.

[18] Li M-c (2021). Lu J, Lu Y, Xiao T-y, Liu H-c, Lin S-q, Xu D, Li G-l, Zhao X-q, Liu Z-g: rpoB Mutations and Effects on Rifampin Resistance in Mycobacterium tuberculosis. Infec Drug Resist.

[19] Miotto P, Cabibbe AM, Borroni E, Degano M, Cirillo DM (2018). Role of disputed mutations in the rpoB gene in interpretation of automated liquid MGIT culture results for rifampin susceptibility testing of Mycobacterium tuberculosis. J Clin Microbiol.

[20] WHO (2021). Catalogue of mutations in Mycobacterium tuberculosis complex and their association with drug resistance.

[21] Gagneux S (2009). Fitness cost of drug resistance in Mycobacterium tuberculosis. Clin Microbiol Infect.

[22] Lin X, Xu Y, Pan X, Xu J, Ding Y, Sun X, Song X, Ren Y, Shan P-F (2020). Global, regional, and national burden and trend of diabetes in 195 countries and territories: an analysis from 1990 to 2025. Sci Rep.

[23] Chihota VN, Niehaus A, Streicher EM, Wang X, Sampson SL, Mason P, Källenius G, Mfinanga SG, Pillay M, Klopper M (2018). Geospatial distribution of Mycobacterium tuberculosis genotypes in Africa. PLoS ONE.

[24] Brynildsrud OB, Pepperell CS, Suffys P, Grandjean L, Monteserin J, Debech N, Bohlin J, Alfsnes K, Pettersson JO-H, Kirkeleite I (2018). Global expansion of Mycobacterium tuberculosis lineage 4 shaped by colonial migration and local adaptation. Science Advances..

[25] Lopez K, Arriaga MB, Aliaga JG, Barreda NN, Sanabria OM, Huang C-C, Zhang Z, García-de-la-Guarda R, Lecca L, Calçada Carvalho AC (2021). Dysglycemia is associated with Mycobacterium tuberculosis lineages in tuberculosis patients of North Lima—Peru. PLoS ONE.

[26] Stucki D, Brites D, Jeljeli L, Coscolla M, Liu Q, Trauner A, Fenner L, Rutaihwa L, Borrell S, Luo T (2016). Mycobacterium tuberculosis lineage 4 comprises globally distributed and geographically restricted sublineages. Nat Genet.

[27] Séraphin MN, Norman A, Rasmussen EM, Gerace AM, Chiribau CB, Rowlinson M-C, Lillebaek T, Lauzardo M (2019). Direct transmission of within-host Mycobacterium tuberculosis diversity to secondary cases can lead to variable between-host heterogeneity without de novo mutation: a genomic investigation. EBioMedicine.

[28] Xu Y, Cancino-Muñoz I, Torres-Puente M, Villamayor LM, Borrás R, Borrás-Máñez M, Bosque M, Camarena JJ, Colomer-Roig E, Colomina J (2019). High-resolution mapping of tuberculosis transmission: Whole genome sequencing and phylogenetic modelling of a cohort from Valencia Region, Spain. PLoS Med.

[29] Comas I, Coscolla M, Luo T, Borrell S, Holt KE, Kato-Maeda M, Parkhill J, Malla B, Berg S, Thwaites G (2013). Out-of-Africa migration and Neolithic coexpansion of Mycobacterium tuberculosis with modern humans. Nat Genet.

[30] Feuerriegel S, Schleusener V, Beckert P, Kohl TA, Miotto P, Cirillo DM, Cabibbe AM, Niemann S, Fellenberg K (2015). PhyResSE: a web tool delineating Mycobacterium tuberculosis antibiotic resistance and lineage from whole-genome sequencing data. J Clin Microbiol.

[31] Miotto P, Tessema B, Tagliani E, Chindelevitch L, Starks AM, Emerson C, Hanna D, Kim PS, Liwski R, Zignol M. A standardised method for interpreting the association between mutations and phenotypic drug resistance in Mycobacterium tuberculosis. Eur Respir J. 2017;50(6):1-13. 10.1183/13993003.01354-2017 PMC5898944 29284687

[32] Coll F, McNerney R, Guerra-Assunção JA, Glynn JR, Perdigão J, Viveiros M, Portugal I, Pain A, Martin N, Clark TG (2014). A robust SNP barcode for typing Mycobacterium tuberculosis complex strains. Nat Commun.

[33] Malm S, Linguissi LSG, Tekwu EM, Vouvoungui JC, Kohl TA, Beckert P, Sidibe A, Rüsch-Gerdes S, Madzou-Laboum IK, Kwedi S (2017). New Mycobacterium tuberculosis complex sublineage, Brazzaville, Congo. Emerg Infect Dis.

[34] Jajou R, Kohl TA, Walker T, Norman A, Cirillo DM, Tagliani E, Niemann S, de Neeling A, Lillebaek T, Anthony RM (2019). Towards standardisation: comparison of five whole genome sequencing (WGS) analysis pipelines for detection of epidemiologically linked tuberculosis cases. Eurosurveillance.

[35] Letunic I, Bork P (2021). Interactive Tree Of Life (iTOL) v5: an online tool for phylogenetic tree display and annotation. Nucleic Acids Res.

